# Transcriptomic Analysis of Pleiotropic Effects of Scandium Chloride on C2C12 Myoblasts

**DOI:** 10.3390/toxics14070623

**Published:** 2026-07-16

**Authors:** Jingyu Zhao, Yingnan An, Jiankai Shi, Libing Ma, Xiaoying He, Ying Liu, Chuncheng Liu

**Affiliations:** Inner Mongolia Key Laboratory of Life Health and Bioinformatics, School of Life Science and Technology, Inner Mongolia University of Science & Technology, Baotou 014010, China; zjy2024023308@stu.imust.edu.cn (J.Z.); anfirst0214@163.com (Y.A.); 18847321854@163.com (J.S.); mlb-xn2004@163.com (L.M.); hxy1124@163.com (X.H.)

**Keywords:** scandium chloride, C2C12, cell proliferation, expression clustering, transcription factor activity

## Abstract

The effects of scandium on skeletal muscle cells remain poorly understood. This study aims to determine whether scandium chloride (ScCl_3_) affects C2C12 myoblast proliferation, and the cellular responses were assessed using CCK-8, flow cytometry, RNA-seq, and bioinformatic analyses after treatment with 0.01 mM and 1 mM ScCl_3_. We verified that 1 mM ScCl_3_ significantly promoted cell proliferation by inducing the G1/S phase transition, while the low concentration had no significant effect. However, GO enrichment analysis failed to capture proliferation-related terms among the top 20 ranked terms. To address this, we grouped genes based on their expression patterns and combined transcription factor activity inference with Hallmark pathway enrichment. This strategy identified distinct functional modules responsive to scandium, involving mitosis, myogenesis, and metabolism, with key regulators including Foxo1, Rela, and myogenic transcription factors. Collectively, our findings suggest that scandium may exert regulatory effects on myoblast proliferation and modulate genes involved in differentiation and metabolism by coordinating multiple functional modules.

## 1. Introduction

Metal elements exhibit dual characteristics in biological systems: essential trace metals regulate oxygen transport and enzyme activation, while toxic heavy metals such as cadmium and lead induce oxidative stress and DNA damage through bioaccumulation [1]. Notably, rare earth elements (REEs) have emerged as novel bioregulators, with studies demonstrating their capacity to enhance cellular proliferation (La^3+^ in hepatocytes) and modulate gene expression (Ce^3+^ in root meristems) [2,3]. However, the tissue-specific mechanisms underlying REEs’ bioactivity remain insufficiently elucidated.

Cerium and lanthanum have been widely studied for their agricultural and biomedical applications. As feed additives, they improve growth performance, nutrient utilization, and immune function in livestock through enhancing digestive enzyme secretion and maintaining intestinal flora homeostasis [4,5,6,7,8,9]. In the field of bone metabolism regulation, lanthanum chloride significantly reduces osteoclast generation by inhibiting the NF-κB signaling pathway [10,11]. Scandium plays a synergistic role with other metals to promote bone regeneration in the form of collagen scaffolds or bone implant materials loaded with trace elements [12]. Among the less explored REEs, scandium is of particular interest: it is the lightest rare earth element with distinct chemical properties, yet its biological effects remain poorly characterized. Recent studies have demonstrated that Sc_2_O_3_ significantly promotes the proliferation of human osteoblast-like cells [13], and the incorporation of scandium and strontium increases osteoblast differentiation in vitro [14]. These findings suggest that scandium possesses osteogenic bioactivity, but whether it exerts direct effects on myoblasts, which share a mesenchymal stem cell origin with osteoblasts, has not been investigated.

Therefore, we treated C2C12 mouse myoblasts with two concentrations of scandium chloride (0.01 mM and 1 mM) and assessed cellular responses through CCK-8, flow cytometry, RNA-seq, and bioinformatic analyses. Our primary objective was to determine whether ScCl_3_ affects myoblast proliferation. In addition, we explored transcriptomic data for potential clues regarding differentiation and atrophy-related gene expression.

## 2. Materials and Methods

### 2.1. Cell Culture

The C2C12 mouse myoblast cell line was purchased from Hunan Fenghui Biotechnology Co., Ltd. (Changsha, China), with a Research Resource Identifier (RRID) of CVCL_0188. The cells were free of mycoplasma contamination. For routine culture, C2C12 cells were maintained in high-glucose DMEM (HyClone, Logan, UT, USA, SH30022.01) supplemented with 10% fetal bovine serum (Gibco, Waltham, MA, USA, 10270106) and 1% penicillin–streptomycin (Solarbio, Beijing, China, P1400) under the conditions of 37 °C and 5% CO_2_ in a humidified incubator (Thermo Scientific, Waltham, MA, USA, model Heracell 150i).

### 2.2. The CCK-8 Experiment

C2C12 cells were seeded into 96-well plates at a density of 5 × 10^3^ cells/well (100 µL/well) and pre-cultured in a 37 °C, 5% CO_2_ incubator for 24 h to ensure adherence. After aspirating the medium, experimental groups were treated with fresh complete medium containing 0.01 or 1 mM scandium chloride hexahydrate (ScCl_3_·6H_2_O; Aladdin, Shanghai, China, S133270), while control groups included blank controls (complete medium without cells) and untreated controls (cells with complete medium, 0 mM ScCl_3_). After 24 h incubation, 10 µL of CCK-8 reagent (SEVEN, Beijing, China, SC119) was added to each well, followed by a 2 h light-protected incubation. Absorbance was measured at 450 nm (primary) and 630 nm (reference) using a Synergy H1 microplate reader (BioTek, Winooski, VT, USA).

Concentration selection: The concentrations of ScCl_3_ (0.01 mM and 1 mM) are supported by recent findings on related lanthanide salts. In mouse L-929 fibroblasts, cell viability for CeCl_3_ and SmCl_3_ decreased significantly only at 1.6 mM (falling below 70% of control), while concentrations below this threshold induced measurable oxidative stress and gene expression perturbations without strong cytotoxicity [15].

### 2.3. Flow Cytometry Analysis

C2C12 cells were seeded into 6-well plates at a density of 2 × 10^5^ cells/well (2 mL/well) and pre-cultured in a 37 °C, 5% CO_2_ incubator for 24 h to ensure complete adherence. After aspirating the original medium, the experimental groups were treated with fresh complete medium containing 0.01 mM or 1 mM ScCl_3_ (2 mL/well), while the control groups were treated with complete medium only.

Following 24 h of incubation, the cells were harvested. The cell pellet was resuspended in 1 mL of pre-cooled 70% ethanol slowly while vortexing gently and then fixed at 4 °C overnight. Before staining, the fixed cells were centrifuged at 300× *g* for 5 min at 4 °C, and the ethanol was removed. The cells were washed twice with 1 mL of PBS and then resuspended in 500 μL of PBS containing 50 μg/mL propidium iodide and 100 μg/mL RNase A. After incubation in the dark at room temperature for 30 min, the samples were analyzed by a BD FACSCalibur flow cytometer. The excitation wavelength of PI was 488 nm, and the emission was detected at 620 nm. At least 20,000 events were collected for each sample, and the cell cycle distribution was analyzed using ModFit LT software (version 6.0).

### 2.4. RNA-Seq Analysis

Sequencing was provided by Gene Denovo Co., Ltd. (Guangzhou, China). Paired-end 150 bp transcriptome sequencing was performed on C2C12 cells from control and scandium-treated groups (0.01 mM or 1 mM ScCl_3_, each containing three biological replicates) using the Illumina HiSeq 2500 platform (San Diego, CA, USA). Raw sequencing data were processed with fastp (version 0.18.0) for quality control with the following parameters: reads containing >5% N bases were discarded; reads with average quality scores below Q20 within a sliding window (size: 4 bp) were filtered; and reads shorter than 50 bp were excluded. Quality-controlled reads were aligned to the mouse reference genome GRCm39 using HISAT2 (version 2.2.1), followed by gene expression quantification with featureCounts (version 2.0.3). Principal component analysis (PCA) was performed on the rlog-transformed expression matrix to assess inter-group variation.

Scatter plot analysis of differentially expressed genes: Differential expression analysis was performed using the limma package (version 3.66.0) for R. DEGs were defined as those with |log2FC| > 1 and adj.P.Val (Benjamini-Hochberg) < 0.05. According to the differential expression pattern under the two treatments, genes were classified into four categories. Common DEGs: differentially expressed in both treatments with |log2FC| > 1; 0.01 mM specific: differentially expressed only at the low concentration; 1 mM specific: differentially expressed only at the high concentration; Non-significant: genes that did not meet the significance threshold in either treatment.

Gene Ontology (GO) enrichment analysis: GO enrichment analysis was performed using the clusterProfiler package (version 4.8.0). The org.Mm.eg.db annotation package (version 3.16.0) was used to map gene symbols to Entrez IDs.

ssGSEA scoring of proliferation-related gene sets: The raw FPKM matrix was filtered and transformed. Gene sets closely related to cell proliferation were selected from the MSigDB Hallmark collection. Single-sample Gene Set Enrichment Analysis (ssGSEA) was performed using the escape package (version 2.6.2) for R to calculate enrichment scores for the above four gene sets in each sample.

Random forest feature importance ranking and grouping: To identify the genes that most contribute to discriminating the 1 mM treatment group from the control, a random forest classifier was trained using the R package randomForest (version 4.7-1.2). The input feature matrix consisted of the log2(FPKM+1) expression values of statistically differentially expressed genes (filtering criterion: adj.P.Val < 0.05, no fold-change restriction). The response variable was the binary group label (control vs. 1 mM treatment). The forest was grown with 500 trees, and gene importance was assessed by the mean decrease in Gini impurity (MeanDecreaseGini). Genes were ranked in descending order of importance and then divided into four consecutive groups (G1 to G4) based on the cumulative distribution of the importance scores.

Gene expression clustering analysis: To explore the expression patterns of genes under different treatment conditions, the average expression value of each gene across the three groups (Control, 0.01 mM ScCl_3_, and 1 mM ScCl_3_) was first calculated from the filtered log2(FPKM+1) expression matrix, resulting in a gene × treatment mean matrix. The expression values of each row were then Z-score normalized to make expression levels comparable across genes. A k-means clustering algorithm (K = 6, Euclidean distance, 25 random starts, maximum iterations 50, random seed 123) was applied to the normalized mean matrix to group genes with similar expression patterns into the same cluster. The expression pattern of each cluster was represented by the average expression value (original log2(FPKM+1) mean) of all genes in that cluster across the three treatment groups.

Transcription factor activity inference. Using the transcription factor–target gene regulatory network provided by the CollecTRI database (mouse version), the ULM (Univariate Linear Model) method from the decoupleR package (version 2.16.0) was applied to infer transcription factor activity scores for the set of statistically differentially expressed genes in each cluster. The ULM method calculates an activity score (positive score indicates activation; negative score indicates repression) by evaluating the enrichment of the statistical metric of each transcription factor’s target genes within the set of statistically differentially expressed genes.

Hallmark pathway enrichment analysis (cluster 1–6): Hypergeometric testing was used to perform Hallmark pathway enrichment analysis on the statistically differentially expressed genes (adj.P.Val < 0.05) within each cluster. The background gene set consisted of all genes in the filtered expression matrix, and the pathway gene sets were derived from the mouse Hallmark gene set collection (50 pathways) in the MSigDB database. Enrichment significance was represented by the *p*-value and transformed into enrichment strength as-log_10_ (*p*_value).

**Figure 1:**
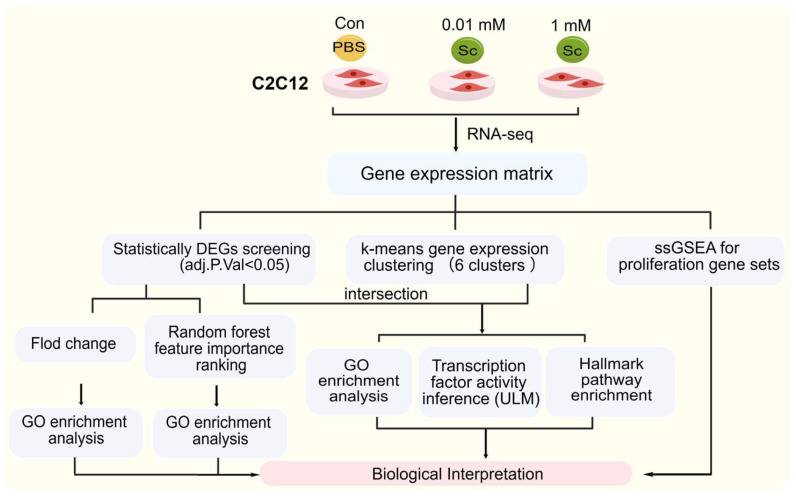
Schematic workflow of the bioinformatics analysis pipeline.

The overall bioinformatics pipeline used in this study is summarized in Figure 1.

### 2.5. RNA Extraction and cDNA Synthesis

C2C12 myoblasts were treated with 0.01 mM and 1 mM ScCl_3_ for 24 h. Total RNA extraction and cDNA synthesis were performed as previously described [16]. Total RNA was isolated using TRIzol reagent (Takara, Kusatsu, Japan, 9109) combined with column purification (Takara MiniBEST Universal RNA Extraction Kit). Reverse transcription was performed in 20 μL reactions containing 5× PrimeScript RT Master Mix, 1 μg RNA, and gDNA Eraser (Takara, RR092A) under the following conditions: 37 °C for 15 min, 85 °C for 5 s. cDNA aliquots were stored at −80 °C.

### 2.6. Real-Time Quantitative PCR (RT-qPCR)

RT-qPCR was conducted using TB Green^®^ Premix Ex Taq™ II (Tli RNaseH Plus) reagent (Takara, RR820A) on a QuantStudio 6 Flex system (Applied Biosystems, Foster City, CA, USA) with the following parameters: 95 °C for 30 s initial denaturation, followed by 40 cycles of 95 °C for 5 s and 60 °C for 30 s. Gapdh was used as an endogenous reference gene. Relative gene expression was calculated via the 2^−ΔΔCt^ method. Each treatment group included three biological replicates. The primer sequences of RT-qPCR are shown in Table S1.

### 2.7. Statistical Analysis

All experimental data were derived from at least three independent biological replicates. Values are presented as mean ± SEM (standard error of the mean). Statistical analyses for CCK-8, flow cytometry, and RT-qPCR were performed using GraphPad Prism (version 9.0.0; GraphPad Software). For comparisons involving the three groups (Control, 0.01 mM ScCl_3_, and 1 mM ScCl_3_), one-way analysis of variance was performed, followed by Tukey’s honest significant difference post hoc test for multiple pairwise comparisons. Significance levels were denoted as * *p* < 0.05 and ** *p* < 0.01.

### 2.8. Generation of Pipeline Graphical Abstract and Workflow

The graphical abstract and workflow of the bioinformatics analysis pipeline were created with BioGDP [17].

## 3. Results

### 3.1. The Effects of ScCl_3_ on the Viability and Cell Cycle Progression of C2C12 Cells

**Figure 2:**
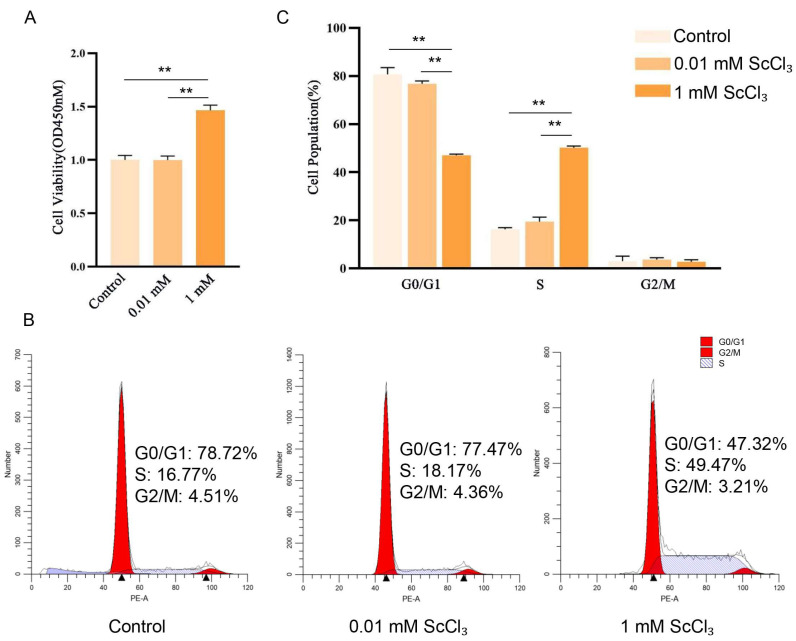
ScCl_3_ affects C2C12 cell viability and cell cycle progression: (**A**) CCK-8 assay showing cell viability changes after 24 h of ScCl_3_ treatment. **: *p* < 0.01, *n* = 6. (**B**) Representative images of flow cytometry analysis of cell cycle distribution after 24 h of ScCl_3_ treatment. (**C**) Quantitative analysis of cell cycle phase distribution (G0/G1, S, G2/M). **: *p* < 0.01, *n* = 3.

We examined the effects of different concentrations of ScCl_3_ (0.01 mM and 1 mM) on cell viability. The CCK-8 assay results showed that 1 mM ScCl_3_ significantly enhanced C2C12 cell viability, whereas the lower concentration of 0.01 mM ScCl_3_ exerted no significant effect (Figure 2A). To investigate how ScCl_3_ affects cell viability, the effects of ScCl_3_ on the cell cycle progression of C2C12 cells were examined using flow cytometry. Results demonstrated that 0.01 mM ScCl_3_ had no significant impact on the cell cycle distribution of C2C12 cells (Figure 2B). In contrast, treatment with 1 mM ScCl_3_ led to a decrease in the number of cells in the G0/G1 phase and an increase in the number of cells in the S phase (Figure 2B,C). These findings suggest that treatment with ScCl_3_ may be associated with the proliferation of C2C12 cells.

### 3.2. Transcriptome Sequencing of C2C12 Cells Treated with ScCl_3_

**Figure 3:**
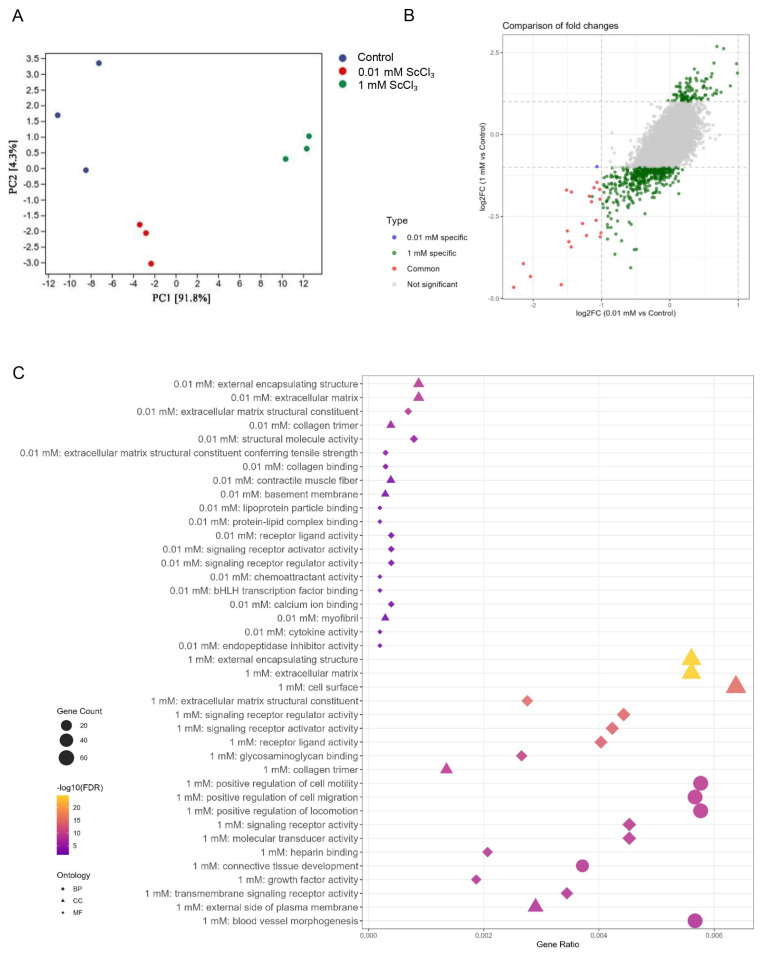
Transcriptome sequencing analysis: (**A**) Principal component analysis (PCA) of gene expression profiles. (**B**) Comparison of gene expression fold changes induced by 0.01 mM and 1 mM treatments. The scatter plot shows the log2FC of all genes in the 0.01 mM vs. Control (*x*-axis) and 1 mM vs. Control (*y*-axis). (**C**) Combined bubble plot of the top 20 GO terms for 0.01 mM and 1 mM ScCl_3_ treatments. The 20 most significant GO terms (by adjusted *p*-value) from each concentration are shown in a single plot (rows 1–20: 0.01 mM ScCl_3_; rows 21–40: 1 mM ScCl_3_).

To more comprehensively illustrate the effects of ScCl_3_ (0.01 mM and 1 mM) on C2C12 cells, particularly its regulatory role in proliferation, we performed RNA-seq (Table S2). PCA showed that the Control, 0.01 mM ScCl_3_, and 1 mM ScCl_3_ groups formed three distinct clusters, and biological replicates within each group were highly consistent (Figure 3A). Next, we screened and analyzed differentially expressed genes (DEGs) (adj.P.Val < 0.05 & |log2FC| > 1) and compared those induced by high and low concentrations of ScCl_3_ (Figure 3B). The results showed that the high concentration induced far more DEGs than the low concentration, and the majority of DEGs induced by the low concentration were contained within those induced by the high concentration. We explored the functions of DEGs induced by low and high concentrations of ScCl_3_ using GO enrichment analysis (Figure 3C, Table S3). However, the top 20 terms (ranked by *p*-value) in the enrichment results for DEGs from both treatments did not contain any proliferation-related terms. For the DEGs induced by the high concentration, the highest-ranked proliferation-related term was only at position 45.

To avoid missing genes with weak expression changes, we then selected statistically differentially expressed genes using only adj.P.Val < 0.05 (without a fold-change threshold) and performed GO enrichment analysis on these genes from both treatment groups (Table S4). Again, the top 20 terms showed no enrichment of proliferation-related terms (Figure S1). We further screened and analyzed genes using adj.P.Val < 0.05 combined with various |log2FC| thresholds, but none of these analyses yielded proliferation-related terms within the top 20 (Table S5).

**Figure 4:**
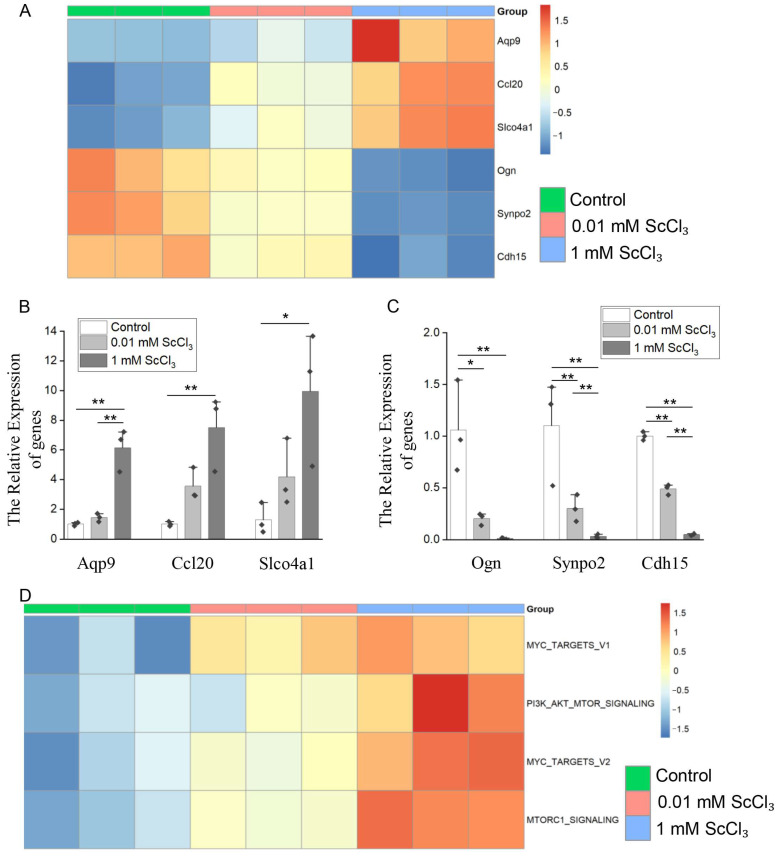
Quantitative validation of RNA sequencing-derived DEGs: (**A**) Cluster heatmap depicting the expression profiles of randomly selected DEGs, including Aqp9, Ccl20, Slco4a1, Ogn, Synpo2, and Cdh15. (**B**,**C**) RT-qPCR verification of six randomly selected DEGs. For each gene, dots represent individual biological samples (n = 3 per group). *: *p* < 0.05, **: *p* < 0.01, *n* = 3. (**D**) ssGSEA heatmap of four proliferation-related gene sets. The heatmap displays ssGSEA enrichment scores for four Hallmark gene sets in mouse samples from the Control, 0.01 mM ScCl_3_ treatment, and 1 mM ScCl_3_ treatment. Colors range from blue (low activity) to red (high activity), representing Z-score-normalized enrichment scores.

Before further exploring why proliferation-related terms were absent from the top 20 of enrichment results, we validated the reliability of the RNA-seq data by RT-qPCR. Six differentially expressed genes induced by 1 mM ScCl_3_ were randomly selected, including three upregulated genes (Aqp9, Ccl20, and Slco4a1) and three downregulated genes (Ogn, Synpo2, and Cdh15). The expression levels of these genes from the transcriptome data (Figure 4A) were consistent with the RT-qPCR results (Figure 4B,C).

### 3.3. Gene Grouping

The above results indicate that the transcriptome sequencing data are reliable. To determine whether stress-responsive genes are associated with cell proliferation, we further calculated the enrichment scores of proliferation-related gene sets (downloaded from the MSigDB Hallmark collection) for each sample using ssGSEA (Figure 4D). Overall, the 1 mM ScCl_3_ treatment exerted the strongest activation of the MYC, PI3K/AKT, and MTORC1 pathways, whereas the low concentration had a weaker effect.

This result indicates that high-concentration ScCl_3_ stress is closely associated with cell proliferation. The rarity of proliferation-related terms among the top 20 or even later terms in GO enrichment analysis can be attributed to the fact that the GO database contains a vast number of terms with highly redundant annotations, which tend to dilute proliferation-related terms. In contrast, a targeted enrichment strategy, particularly the ssGSEA, can effectively capture the relevance of cell proliferation.

Stress typically induces synergistic perturbations across multiple pathways, and relatively mild biological signals such as cell proliferation may be masked. Clearly, scandium exerts pleiotropic effects. To reveal the multiple pathways affected by scandium, we attempted to classify these genes based on specific features. This would enable a more comprehensive understanding of scandium’s actions, rather than merely demonstrating its association with proliferation.

**Figure 5:**
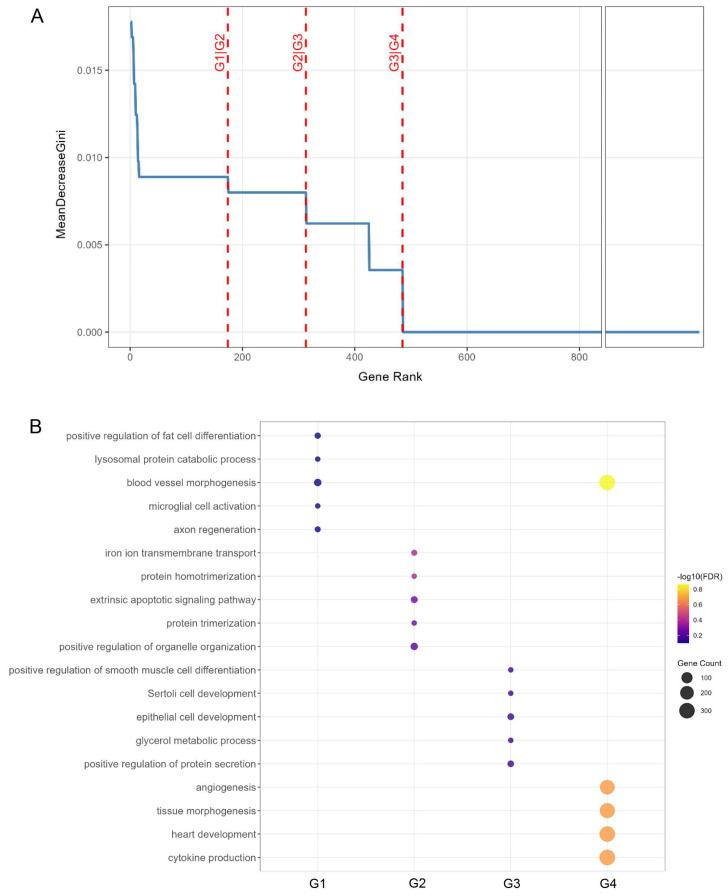
Random forest-based gene grouping and functional heterogeneity: (**A**) Random forest importance plot of statistically differentially expressed genes. Genes are ordered from left to right by decreasing mean decrease in Gini (MeanDecreaseGini). The blue line connects the MeanDecreaseGini values of individual genes. Vertical red dashed lines mark the boundaries of the four importance groups: G1 (ranks 1–174), G2 (175–313), G3 (314–485), and G4 (486 to the end). The x-axis is broken between ranks 800 and 5800. (**B**) Bubble plot of GO biological process enrichment for random forest importance groups. GO BP enrichment analysis was performed separately for each of the groups G1–G4, and the top 5 most significant terms per group are displayed.

We trained a random forest classifier to calculate the importance of statistically differentially expressed genes (1 mM ScCl_3_ vs. Control) according to the group assignment using the mean decrease in Gini impurity. Based on the importance scores, the genes were partitioned into four consecutive groups (Figure 5A). Subsequently, we performed GO biological process enrichment analysis for each group. The top 5 most significant terms per group were used to generate a bubble plot (Figure 5B). The results showed that across all groups G1 to G4, the enriched terms involved processes such as adipocyte differentiation, microglial activation, etc., but none of the terms were directly related to proliferation. Moreover, the enriched terms within each group were functionally diverse and did not form functionally coherent modules. Thus, although random forest could distinguish genes based on their contribution to classification, the grouping results exhibited high heterogeneity in terms of biological functions.

**Figure 6:**
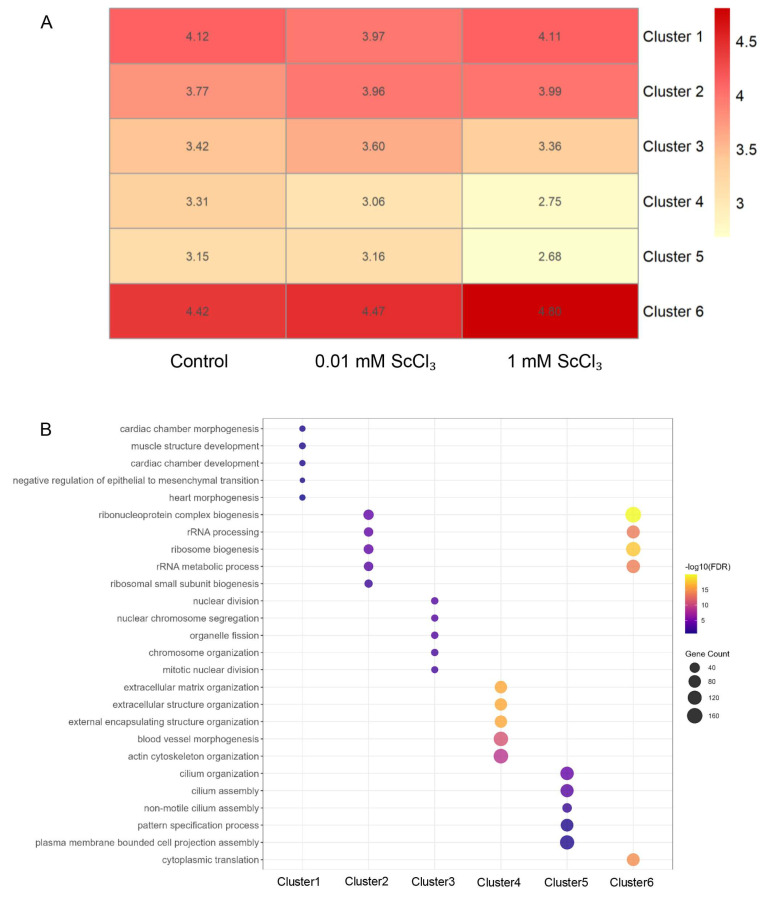
Expression pattern clustering and cluster-specific GO enrichment: (**A**) Heatmap of average expression patterns of the six clusters. After Z-score normalization of the log2(FPKM+1) mean matrix, k-means (K = 6) clustering was applied to partition the genes into six clusters. The heatmap displays the average expression value (original log2(FPKM+1) mean) of all genes in each cluster across the three groups (Control, 0.01 mM ScCl_3_, 1 mM ScCl_3_). Color from light yellow (low) to red (high) indicates expression level, and the numerical values are shown inside the cells. Rows are arranged by cluster number (Cluster 1–6), and columns are ordered by treatment group. (**B**) Bubble plot of the top five GO biological process terms for each cluster. GO BP enrichment analysis was performed separately on the statistically differentially expressed genes within each of the six clusters. The top 5 significant terms are displayed for each cluster.

We then attempted to group genes according to their expression patterns. Using the k-means clustering algorithm on the normalized mean matrix, we divided the genes into six clusters (Figure 6A). For each expression cluster, we extracted the statistically differentially expressed genes (1 mM ScCl_3_ vs. Control) and conducted GO biological process enrichment analysis. The top 5 enriched terms per cluster are shown in Figure 6B. The results showed that the enriched terms were highly concentrated within each cluster: Cluster 1 was involved in heart/muscle development; both Cluster 2 and Cluster 6 were enriched in ribosome biogenesis and translation processes, with Cluster 6 exhibiting stronger significance; Cluster 3 regulated mitosis; Cluster 4 participated in extracellular matrix organization and angiogenesis; and Cluster 5 was responsible for cilium assembly and cell polarity formation.

### 3.4. Analysis of the Regulatory Network of ScCl_3_

The above results showed that genes with similar expression patterns tended to be enriched in distinct biological processes, suggesting that they might be co-regulated by common transcriptional regulators in response to scandium treatment. To explore this possibility and to gain insight into the molecular mechanisms by which ScCl_3_ alters gene expression and influences these biological processes, we next inferred transcription factor (TF) activities in each cluster using the statistically significant differentially expressed genes within the cluster.

**Figure 7:**
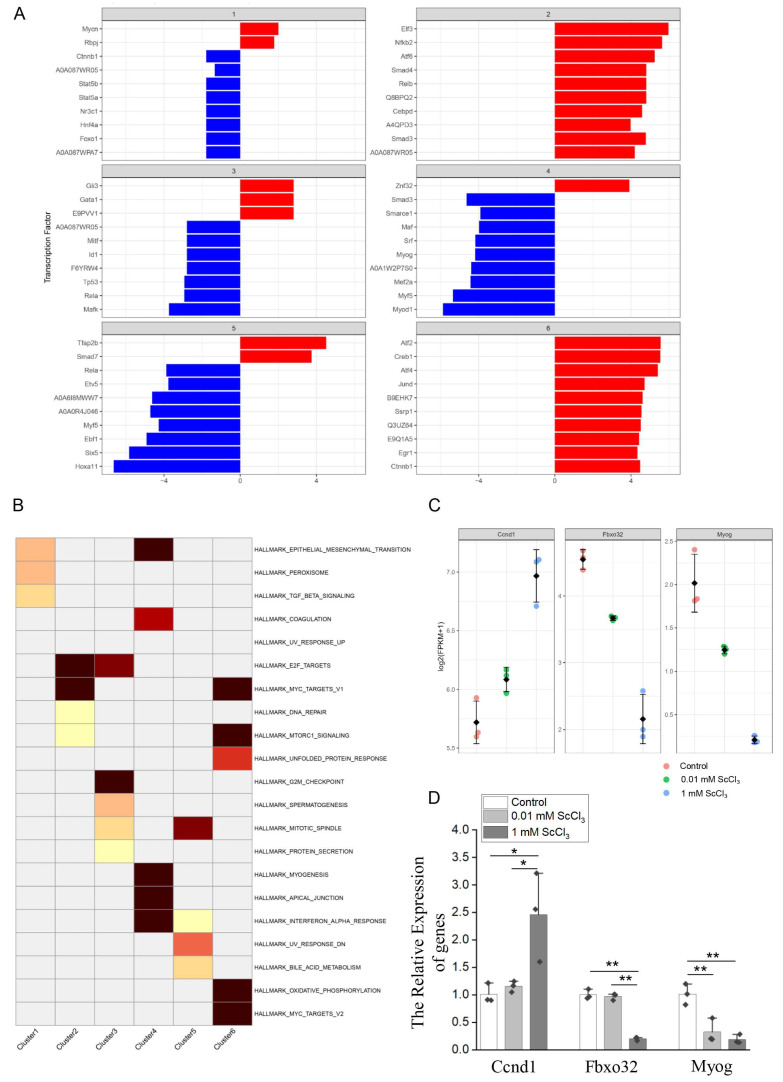
Transcription factor activity and validation of key factors: (**A**) Top 10 transcription factors with the highest absolute activity scores in each cluster. Transcription factor activity scores were calculated using the CollecTRI regulatory network and the ULM method based on the statistically differentially expressed genes within each cluster. The y-axis shows the transcription factor names (ordered by descending score), and the x-axis shows the activity score. Red bars indicate activation (positive score), and blue bars indicate repression (negative score). Panels are arranged by Cluster 1–6, with independent sorting within each cluster. (**B**) Heatmap of the top 5 Hallmark pathways enriched in each cluster. Hallmark pathway hypergeometric enrichment analysis was performed on the statistically differentially expressed genes within each cluster, and the top 5 pathways with the highest enrichment significance were selected for each cluster. Color from light yellow (low) to dark red (high) indicates increasing enrichment significance. (**C**) The expression levels of Fbxo32, Ccnd1, and Myog after ScCl_3_ treatment (log_2_(FPKM+1)). This panel is based on transcriptome sequencing data. For each gene, dots represent individual biological samples (*n* = 3 per group); bars and error bars indicate group means ± SD, respectively. (**D**) Validation of expression changes of Fbxo32, Ccnd1, and Myog by RT-qPCR. For each gene, dots represent individual biological samples (*n* = 3 per group). *: *p* < 0.05, **: *p* < 0.01, *n* = 3.

Transcription factor activity was inferred using the CollecTRI network and the ULM method, and the top 10 transcription factors with the highest absolute activity scores in each cluster are shown (Figure 7A). The results showed that the active transcription factors in each cluster were highly consistent with their functional enrichment profiles (Figure 6B). For example, in Cluster 3 (mitosis and chromosome segregation), the most active transcription factors included Gli3, Gata1, Mitf, Id1, Tp53, Rela, and Mafk, among others. Among these, Tp53 and Rela are core regulators of the cell cycle and DNA damage response. Cluster 4 also drew our attention, as it was significantly enriched in myogenic regulatory factors, including Myog, Myod1, Mef2a, Myf5, etc. This feature is consistent with the myoblast background of the cells and also suggests that ScCl_3_ treatment may affect myogenic differentiation.

Based on the identification of key transcription factors, we performed ssGSEA enrichment analysis using the Hallmark gene sets to better establish the correlations between these transcription factors and biological processes. The top 5 pathways with significant enrichment scores are displayed for each group (Figure 7B).

GO enrichment of Cluster 1 showed its primary involvement in cardiac/muscle development and morphogenesis. One of the transcription factors with the highest activity in this cluster was Foxo1 (negative score). Foxo1 is a key regulator of muscle development, metabolism, and atrophy, and it can inhibit the mTOR signaling pathway—a pathway identified as significantly enriched in Cluster 1 by ssGSEA. Analysis of differentially expressed genes in the 1 mM ScCl_3_ treatment group identified Fbxo32 (Figure 7C), a direct target of Foxo1. We further validated the expression of Fbxo32 and confirmed that it was indeed downregulated (Figure 7D).

GO enrichment of Cluster 3 showed its involvement in mitosis, nuclear division, and chromosome segregation. Hallmark pathways concurrently enriched included E2F targets, the G2/M checkpoint, and the mitotic spindle. Transcription factor activity analysis placed Rela (NF-κB subunit) within the top 10 of Cluster 3. Rela is known to bind directly to the NF-κB site in the promoter of Ccnd1 (cyclin D1), promoting G1/S transition, and Ccnd1 transcript levels were elevated in the 1 mM group (Figure 7C). To test this regulatory hypothesis, we examined the expression change of Ccnd1 by RT-qPCR and found that its expression was increased (Figure 7D).

Cluster 4 transcription factor activity analysis revealed a strong enrichment of myogenic regulatory factors (Myog, Myod1, Mef2a, Myf5, etc.), and Hallmark pathway analysis identified MYOGENESIS. Therefore, we selected Myog (myogenin), the master transcription factor of terminal skeletal muscle differentiation, as a validation target and examined its expression level (Figure 7C,D). Consistent with the transcriptome data, RT-qPCR results showed that Myog expression was downregulated upon scandium chloride treatment.

## 4. Discussion

In this study, we demonstrated that 1 mM ScCl_3_ promotes C2C12 myoblast proliferation by inducing G1/S transition, while transcriptomic analysis further revealed distinct functional modules suggesting potential influences on myogenic differentiation and metabolism. Conventional GO enrichment analysis of the full DEG set failed to capture proliferation-related signals among the top-ranked terms. A previous study found that intermittent hypoxia-induced DEGs in the mouse hippocampus did not enrich for nerve injury terms in standard GO analysis, but genes associated with stress duration successfully captured neurodegeneration-related pathways [18]. In the present study, we first grouped genes based on their expression patterns and then performed functional enrichment on the statistically differentially expressed genes within each group. This strategy effectively separated genes with distinct functional responses and revealed the pleiotropic effects of scandium.

Cluster 3 was associated with proliferation. Transcription factor analysis placed Rela (NF-κB subunit) among the top regulators. Notably, NF-κB directly binds the Ccnd1 promoter [19], and Ccnd1 expression was indeed elevated upon ScCl_3_ treatment. Rare earth elements, including lanthanum, have been shown to modulate NF-κB activity [11], and our findings extend this regulatory potential to scandium in skeletal muscle cells. Cluster 4 transcription factor activity analysis revealed a strong enrichment of myogenic regulatory factors, including Myog, Myod1, Mef2a, and Myf5. RT-qPCR confirmed that *Myog*, the master regulator of terminal skeletal muscle differentiation [20], was significantly downregulated upon ScCl_3_ treatment. This result suggests that scandium may simultaneously suppress differentiation capacity while promoting proliferation, a well-known inverse relationship in muscle biology [21].

Notably, previous studies have demonstrated that scandium-containing materials promote osteoblast differentiation [13,14], yet our findings suggest that the effect of scandium on myogenic differentiation may be opposite, highlighting the importance of lineage-specific responses. Additionally, our clustering-based strategy, combined with TF activity inference and Hallmark enrichment, provides a useful framework for dissecting pleiotropic stress responses. This analytical approach provides deeper causal clues than single enrichment analysis.

Several limitations should be acknowledged. First, our observations on myogenic differentiation and atrophy-related metabolism are based solely on transcriptomic data and require functional validation (e.g., differentiation assays, protein degradation measurements). Second, the inferred activities of Rela, Foxo1, and myogenic TFs need direct experimental confirmation. Third, only a single time point (24 h) was examined, and the temporal dynamics of ScCl_3_-induced changes remain unexplored.

Conclusions. In summary, this study reveals that 1 mM ScCl_3_ promotes C2C12 myoblast proliferation through cell-cycle-related pathways involving Rela and Ccnd1. Transcriptomic analysis further identified distinct functional modules suggesting potential modulation of myogenic differentiation and metabolism. This strategy effectively separated genes with distinct functional responses and revealed the pleiotropic effects of scandium. Our work provides new insights into the mechanism of action of rare earth elements in skeletal muscle cells and offers a reference analytical framework for deep mining of transcriptome data under other stress conditions.

## Data Availability

The data presented in this study are openly available in FigShare at https://doi.org/10.6084/m9.figshare.32974835. Supplementary Materials (Tables S1–S5) are also included with this article.

## Supplementary Materials

The following supporting information can be downloaded at https://www.mdpi.com/article/10.3390/toxics14070623/s1, Table S1: Primer sequences used for RT-qPCR validation. Table S2: Gene expression matrix. Table S3: Complete list of Gene Ontology (GO) enrichment results for differentially expressed genes induced by 0.01 mM and 1 mM ScCl_3_ (|log2FC| > 1, adj.P.Val < 0.05). BP, biological process; CC, cellular component; MF, molecular function. Table S4: Complete list of Gene Ontology (GO) enrichment results for statistically differentially expressed genes identified using adj.P.Val < 0.05 without a fold-change threshold. This analysis was performed to avoid missing genes with weak expression changes. Table S5: Gene Ontology (GO) enrichment results for differentially expressed genes identified using adj.P.Val < 0.05 combined with various |log2FC| thresholds (|log2FC| > 0.75 and 0.8). Figure S1. Bubble plot of top 20 GO terms for 0.01 mM and 1 mM ScCl_3_ treatments (adj.P.Val < 0.05, no fold-change threshold).
