# Supplementary material for: Transcriptomic Analysis of Pleiotropic Effects of Scandium Chloride on C2C12 Myoblasts
Source: Toxics. 2026 Jul 16;14(7):623. doi: 10.3390/toxics14070623 (PMC13417790; doi:10.3390/toxics14070623)
Supplement: Supplementary file 1 [file toxics-14-00623-s001.zip › Figure S1.pdf]

Transcriptomic Analysis of Pleiotropic Effects of Scandium Chloride on C2C12 Myoblasts

Jingyu Zhao[*], Yingnan An[*], Jiankai Shi, Libing Ma, Xiaoying He, Ying Liu[#], Chuncheng Liu[#]

Inner Mongolia Key Laboratory of Life Health and Bioinformatics, School of Life Science and Technology, Inner Mongolia University of Science & Technology, Baotou, 014010, China

*These authors contributed equally to this work.

[#]Correspondence Author

Ying Liu, Email: liuying1529@163.com

Chuncheng Liu, Email: liuchuncheng.china@gmail.com

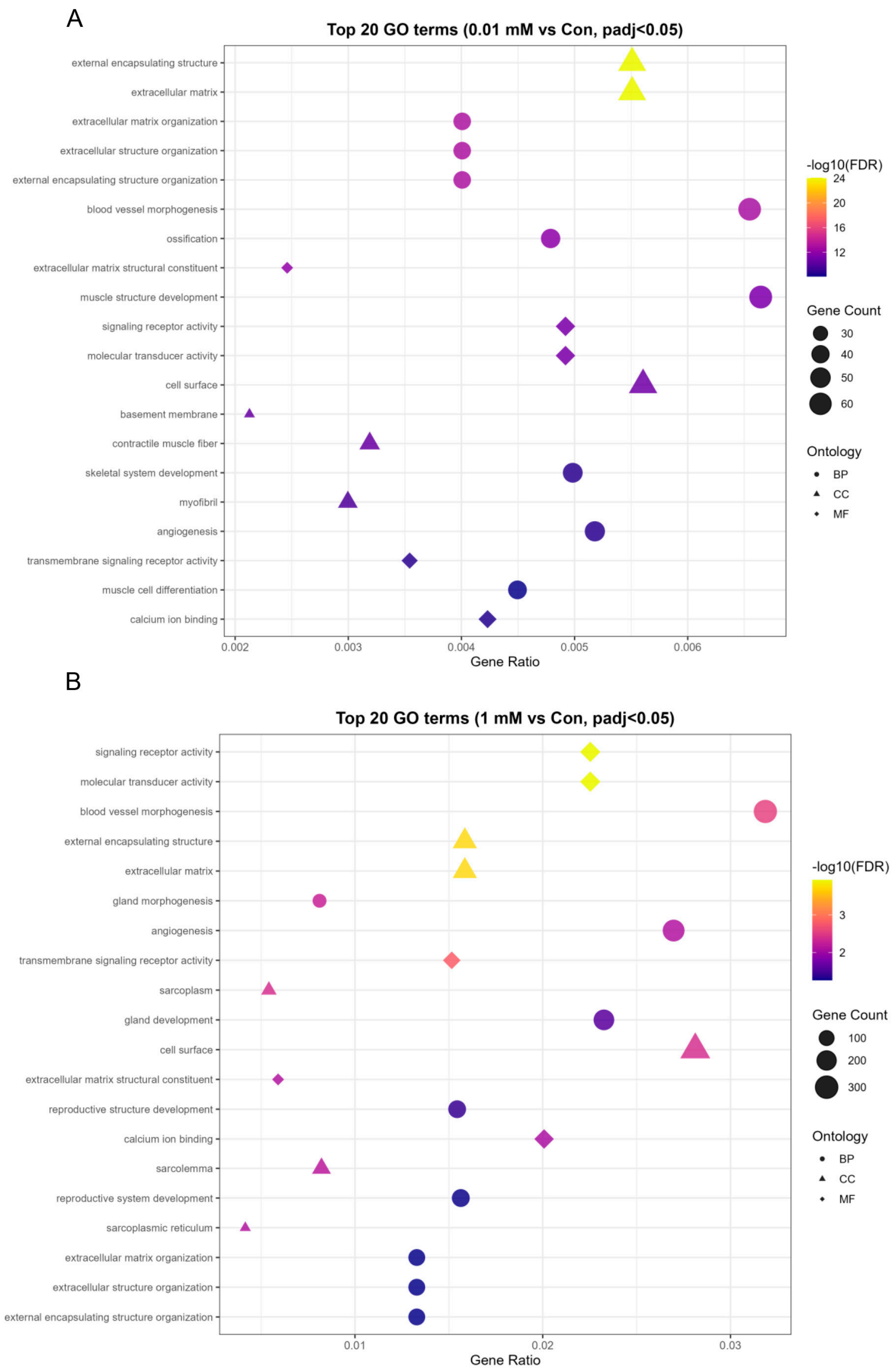

Supplementary Figure S1. Bubble plot of the top 20 GO terms: (A) Bubble plot of the top 20 GO terms enriched in the 0.01 mM $ScCl_3$ treatment. (B) Bubble plot of the top 20 GO terms enriched in the 1 mM $ScCl_3$ treatment. The 20 most significant GO terms (by adj.P.Val) from the combined

analysis of Biological Process (BP), Cellular Component (CC), and Molecular Function (MF) are shown. The x-axis represents the Gene Ratio Point size is proportional to the number of differentially expressed genes (Count). Point color indicates enrichment significance −log10(adj.P.Val).
